# Enhancing Oil Recovery from Low-Permeability Reservoirs with a Thermoviscosifying Water-Soluble Polymer

**DOI:** 10.3390/molecules26247468

**Published:** 2021-12-09

**Authors:** Xiaoqin Zhang, Bo Li, Feng Pan, Xin Su, Yujun Feng

**Affiliations:** 1 Exploration & Development Research Institute, Daqing Oilfield Company of PetroChina, Daqing 163712, China; zhangxiaoqin@petrochina.com.cn (X.Z.); libo123@petrochina.com.cn (B.L.); panfeng1985@petrochina.com.cn (F.P.); 2 Polymer Research Institute, State Key Laboratory of Polymer Materials Engineering, Sichuan University, Chengdu 610065, China; xinsu@scu.edu.cn

**Keywords:** water-soluble polymer, thermoviscosifying polymer, smart polymer, enhanced oil recovery, polymer flooding

## Abstract

Water-soluble polymers, mainly partially hydrolyzed polyacrylamide (HPAM), have been used in the enhanced oil recovery (EOR) process. However, the poor salt tolerance, weak thermal stability and unsatisfactory injectivity impede its use in low-permeability hostile oil reservoirs. Here, we examined the adaptivity of a thermoviscosifying polymer (TVP) in comparison with HPAM for chemical EOR under simulated conditions (45 °C, 4500 mg/L salinity containing 65 mg/L Ca^2+^ and Mg^2+^) of low-permeability oil reservoirs in Daqing Oilfield. The results show that the viscosity of the 0.1% TVP solution can reach 48 mPa·s, six times that of HPAM. After 90 days of thermal aging at 45 °C, the TVP solution had 71% viscosity retention, 18% higher than that of the HPAM solution. While both polymer solutions could smoothly propagate in porous media, with permeability of around 100 milliDarcy, TVP exhibited stronger mobility reduction and permeability reduction than HPAM. After 0.7 pore volume of 0.1% polymer solution was injected, TVP achieved an incremental oil recovery factor of 13.64% after water flooding, 3.54% higher than that of HPAM under identical conditions. All these results demonstrate that TVP has great potential to be used in low-permeability oil reservoirs for chemical EOR.

## 1. Introduction

Crude oil is not only a leading fuel source, but also provides feedstocks for synthetic polymers. Some of the polymers, mainly the water-soluble ones, are used in turn in various phases of oil production [1], especially in the so-called enhanced oil recovery (EOR) or tertiary oil recovery process [2] to sustain a continuous supply of energy. Currently, partially hydrolyzed polyacrylamide (HPAM) tops the list of water-soluble polymers (WSPs) used in chemical EOR [3]. However, the poor salt tolerance and weak thermal stability, as well as the unsatisfactory injectivity of high-molecular-weight HPAM, impede its wider use in harsh and low-permeability oil reservoirs. Therefore, novel WSPs with improved properties, such as thermoviscosifying polymers (TVPs) [4], show strong potential for use in the EOR industry.

After water flooding, there is still a considerable amount of oil remaining as it is not swept away owing to the “viscous fingering” effect resulted from the low viscosity of the displacing fluid [5]. In other words, the driving water moves more easily than the displaced oil, leading to the unfavorable mobility ratio between the water and oil phases, reducing the areal sweep efficiency during the secondary oil recovery process. To decrease the mobility ratio to desirable values, generally less than unity, a high-molecular-weight WSP, normally HPAM, is added to thicken the chasing fluid, rendering a stable, uniform front and reducing the water permeability, thus decreasing its mobility and diverting the following driving fluid into less permeable zones to sweep away more oil [6]. Since the early 1960s, HPAM has been successfully used in a series of chemical EOR projects [7]. However, the anionic polyelectrolyte feature of HPAM has limited its use in saline and hard water, because the Coulombic repulsion among carboxylate groups would be screened by the inorganic cations, such as Na^+^ and K^+^, present in the connate water or the produced fluid used for preparing polymer solutions [8], causing the relative extended conformation of polymer chains to adopt a more compact state, decreasing the hydrodynamic volume of the polymer coils, and finally deteriorating the viscosity buildup [9]. If divalent cations such as Ca^2+^ and Mg^2+^ are co-present in the brine, the complexation between these alkaline earth metal ions and carboxylate groups may bring about the precipitation and phase separation of HPAM from the polymer solution [10,11], completely eliminating the thickening power of the displacing fluid. When used in high-temperature oil reservoirs, the thickening power of the HPAM solution will be diminished as the viscosity of the polymer solution decreases upon increasing temperature. In addition, the side amide groups along the HPAM skeleton undergo extensive hydrolysis, resulting in more carboxylate moieties that further interact with the polyvalent cations, seriously weakening the long-term thermal stability of the polymers used [12].

Fortunately, the great success of polymer EOR has been witnessed in Daqing Oilfield owing to its mild oil reservoir conditions. As early as in 1965, Daqing started polymer screening and initiated a feasibility study of polymer flooding. Seven years later, a pilot test of polymer flooding was implemented. In 1991, an extended field trial was carried out, and in 1995, scaled-up polymer flooding was put into effect. To date, around 14% of original oil in place (OOIP) from polymer EOR has been obtained. A laboratory study of another polymer-based EOR mode, alkali/surfactant/polymer (ASP), was initiated in 1972 in Daqing, and then a pilot test, field trial, and scaled-up field application were carried out in 1994, 2000, and 2014, respectively; approximately 20% of OOIP over water flooding was attained by such a ternary formulation. By 2019, the accumulated incremental oil production by both polymer and ASP flooding had reached 265 million tons in Daqing Oilfield, and more than 10 million tons of oil was produced annually for a continuous period of 18 years. In 2019, the total incremental oil production by polymer and ASP flooding reached 10.4 million tons, of which polymer flooding alone represented 6.03 million tons, supporting the sustainable development of the mature Daqing Oilfield.

Regarding the extension of chemical EOR, however, Daqing Oilfield is still facing some challenges, one of which is the lower recovery factor from the type III oil reserve, where the average permeability is less than 100 milliDarcy (mD). While the crude reserve amounts to 1.86 billion tons, the recovery factor of pilot polymer flooding projects in such low-permeability oil layers ranges from only 4% to 10.2% (OOIP). Such relatively low recovery factors cannot justify economically the investment in chemical EOR. Furthermore, the polymer mother solution is prepared practically at the surface with relatively less saline fresh river water, and then diluted with more saline produced fluids. Such an operation process results in two requirements for EOR polymers: first, the viscosity of the polymer solution prepared with currently used, commercial, high-molecular-weight HPAM must generally be high in order to ensure sufficient remaining viscosity when pumped into the relative high-temperature target oil layers underground, unavoidably increasing the burden of the pumps on the surface; second, the polymer used must be salt-tolerant as it must maintain sufficient viscosity after dilution with the more saline produced fluid or contact with the connate water. Therefore, polymer solutions with lower viscosity on the surface but with elevated thickening power in the porous media are highly desirable to be developed for such low-permeability oil reservoirs.

In an endeavor to address the thermothinning defect of HPAM, thermothickening or thermoviscosifying polymers (TVPs) were pioneered by Hourdet and his coworkers [13] in the early 1990s. They incorporated some “blocks” or “grafts” with the characteristic of a lower critical solution temperature (LCST) onto the backbone of a WSP. In the semi-dilute region and when above the LCST, such thermo-sensitive pendant groups cluster intermolecularly together to form three-dimensional transient networks, promoting a viscosity enhancement macroscopically. However, the molecular weight of the early reported TVPs is generally less than half a million; thus, much higher polymer concentrations are needed to satisfy the thickening requirement, which is currently economically infeasible for the end uses. In addition, some inorganic salts, mainly K_2_CO_3_, are always needed to tune the thermoassociation temperature of TVPs that contain poly(ethylene oxide) (PEO) as side chains [13,14,15,16,17]. These drawbacks limit the large-scale manufacturing of TVPs and hinder the acceptance of petroleum engineers, who always desire less expensive, low-dosage, and high-molecular-weight WSPs for EOR use. To address these defects, our laboratory has been continuously involved in improving the TVPs by increasing the molecular weight with the copolymerization of acrylamide and a macromonomer [18,19,20,21,22], and improving the hydrosolubility with the inverse emulsion or dispersion polymerization of Pluronics with acrylamide and sodium acrylate [23,24,25].

To solve the poor injectivity of currently used high-molecular-weight HPAM and to improve the salt tolerance for the polymers used in the type III oil layers of Daqing Oilfield, which benefit from the low viscosity at lower temperatures of the TVPs, in this work, we attempt to check the adaptivity of our laboratory-prepared TVP polymer for the type III oil reserve of Daqing Oilfield. The thickening power in both synthetic fresh brine and synthetic produced fluids, the temperature dependence of the viscosity, the long-term thermal stability, transportation properties, and enhanced oil recovery efficiency of the TVP were examined in comparison with currently used HPAM with a similar molecular weight.

## 2. Results and Discussion

### 2.1. Concentration Dependence of Thickening Power

As for its use in the chemical EOR process, the primary criterion is the thickening power of a polymer solution at low concentrations. To simulate the injection process in Daqing Oilfield, i.e., the preparation of polymer mother solutions with fresh surface water, but diluted with produced saline fluid, the thickening power of both TVP and HPAM in synthetic fresh surface river water (950 mg/L NaCl) or synthetic produced fluid (4435 mg/L NaCl + 65 mg/L Ca^2+^ and Mg^2+^) was compared.

**Figure 1:**
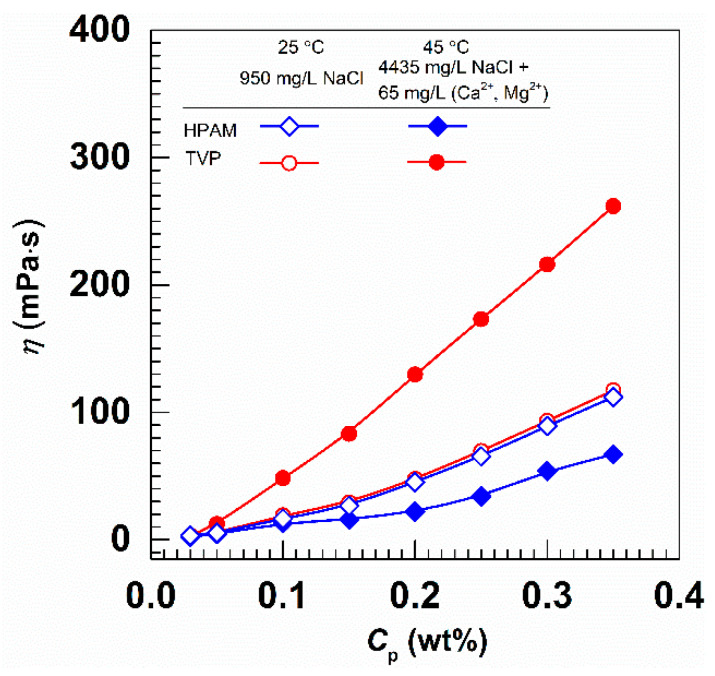
Concentration dependence of TVP versus HPAM at simulated surface (25 °C, 950 mg/L NaCl) and oil reservoir conditions of Daqing Reserve III (45 °C, 4435 mg/L NaCl + 65 mg/L Ca^2+^ + Mg^2+^). Shear rate, 7.34 s^−1^.

As displayed in Figure 1, one can find that the apparent viscosity (*η*) of all polymer solutions increases with increasing polymer concentration, regardless of the salinity and temperature. For example, at 25 °C and in synthetic surface water, the viscosity is elevated from around 1 mPa·s at 0.04% to 100 mPa·s at 0.35%, a one-hundred-times enhancement. Under the same conditions, the thickening power of TVP is almost the same as that of HPAM, as confirmed by the superimposed viscosity plots (the open symbols in Figure 1).

In stark contrast, however, at 45 °C and in the synthetic produced fluid, the viscosity of the HPAM solutions drops to nearly half that at 25 °C and in the synthetic surface water, indicative of their poor salt tolerance and thermal stability. On the contrary, under this more hostile environment, TVP shows an abnormal thickening ability. For instance, compared to the case at 25 °C, the viscosity of the 0.1% TVP solution triples; similarly, at 0.35%, the viscosity increases from approximately 110 mPa⋅s to 270 mPa⋅s, two-times more for the viscosity increment. Note that at the practically used polymer concentration (0.1%), while they both have very close viscosity values at surface conditions (25 °C, 950 mg/L NaCl), TVP has five-times-higher viscosity than HPAM under the simulated oil reservoir conditions (45 °C, 4500 mg/L TDS). These results demonstrate that TVP has much stronger thermothickening power than HPAM under the condition of the type III oil layers in Daqing Oilfield.

### 2.2. Effect of Temperature on Polymer Solution Viscosity

The pumping process of a polymer solution into oil-bearing zones downhole experiences temperature variation—that is, a change from the lower temperature at the surface to a higher temperature in the formation. To simulate this temperature variation procedure, here, we examined the viscosity change during temperature scanning from 20 to 70 °C for 0.1% TVP and HPAM solutions with the abovementioned two salinities.

**Figure 2:**
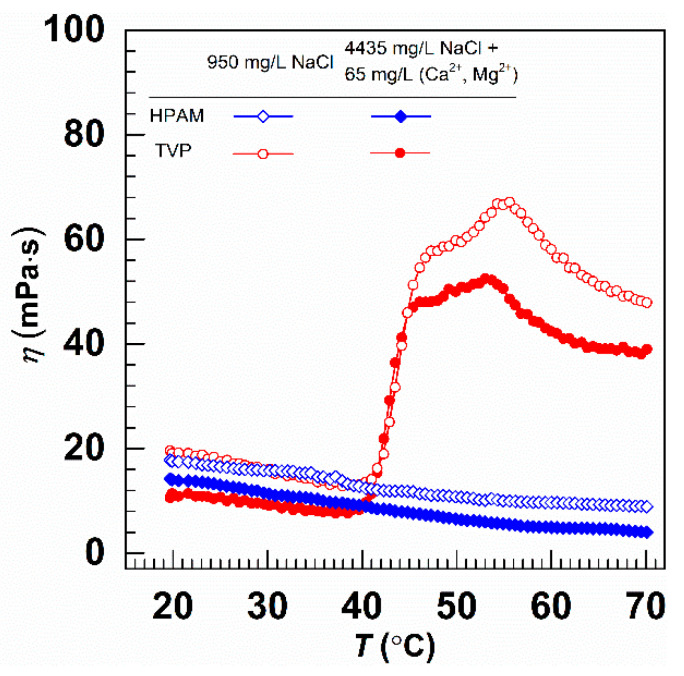
Viscosity changes during temperature scan for 0.1% TVP or HPAM solution in the presence of 950 mg/L NaCl or 4500 mg/L TDS with 65 mg/L Ca^2+^ and Mg^2+^. Shear rate, 7.34 s^−^^1^; heating rate, 2 °C/min.

As exhibited in Figure 2, irrespective of the salinity, HPAM solutions continuously become thinned upon increasing temperature from 20 to 70 °C, and the viscosity in the synthetic river water is always higher than that in the more saline produced fluid.

On the contrary, the viscosity of the 0.1% TVP solution with 950 mg/L NaCl decreases from 20 mPa⋅s at 20 °C to around 16 mPa⋅s when the temperature reaches 40 °C, after which the viscosity sharply increases to 60 mPa⋅s at 45 °C, three times that at 20 °C. When the temperature is further increased to 70 °C, the viscosity rises to a peak of 68 mPa⋅s at around 55 °C, finally descending to 50 mPa⋅s at 70 °C. In the presence of 4500 mg/L TDS containing 65 mg/L Ca^2+^ and Mg^2+^, the TVP solution shows a similar trend to that in 950 mg/L NaCl; the viscosity is initially 11 mPa⋅s at 20 °C, then smoothly descends to 10 mPa⋅s at 40 °C, then rises to around 50 mPa⋅s at 45 °C, and finally settles at 40 mPa⋅s at 70 °C. In both salinity modes, the TVP solutions show a significant thermoviscosifying response, and the viscosity at target reservoir conditions is 3−5 times that at the surface, highly superior to that of the HPAM solutions.

**Figure 3:**
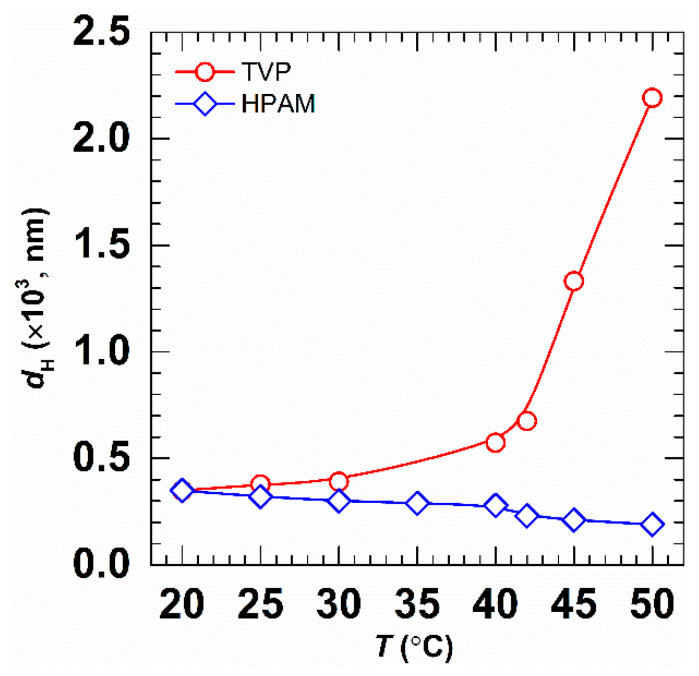
The hydrodynamic size (*d*_H_) of both TVP and HPAM plotted against temperature for 0.1% polymer solution. Solvent: synthetic produced fluid, 4500 mg/LTDS containing 65 mg/L Ca^2+^ and Mg^2+^.

To unravel the mechanisms behind the thermothickening of TVP and the thermothinning properties of HPAM, the size of the hydrated polymer coils, i.e., the hydrodynamic diameter (*d*_H_) of the polymer chains, measured by dynamic light scattering, is plotted against temperature for 0.1% TVP or HPAM solution. As can be seen from Figure 3, the *d*_H_ of the 0.1% HPAM solution constantly decreases from 350 to 200 nm when increasing the temperature from 20 to 50 °C, in good agreement with the continuous viscosity drop during the temperature scan (Figure 2). On the contrary, the size of the TVP polymer smoothly increases from 350 to 550 nm when the temperature is increased from 20 to 40 °C. If we further increase the temperature to 45 °C, the size becomes 1400 nm, and it reaches 2200 nm at 50 °C, implying that intermolecular clusters are formed, corresponding well with the macroscopic viscosity enhancement upon increasing temperature (Figure 2). These results indicate that the thermothickening behavior of the TVP solutions can be ascribed to the thermo-association of the polyether grafts.

### 2.3. Shear Rate Dependence of Polymer Solution Viscosity

Generally, EOR polymer solutions exhibit shear thinning or pseudo-plastic behaviors, i.e., the viscosity of the polymer solution decreases upon increasing shear rate, as the polymer coils become extended when shear force is applied. The viscosity of such non-Newtonian fluids near the wellbore is lower due to the high pumping rate and thus high shear rate, favoring the injectivity; once the polymer slug moves far into the reservoir, the shear rate declines and the viscosity increases, providing desirable mobility control [26]. Therefore, it is necessary to check if the TVP polymer used in this work exhibits similar shear thinning to HPAM.

**Figure 4:**
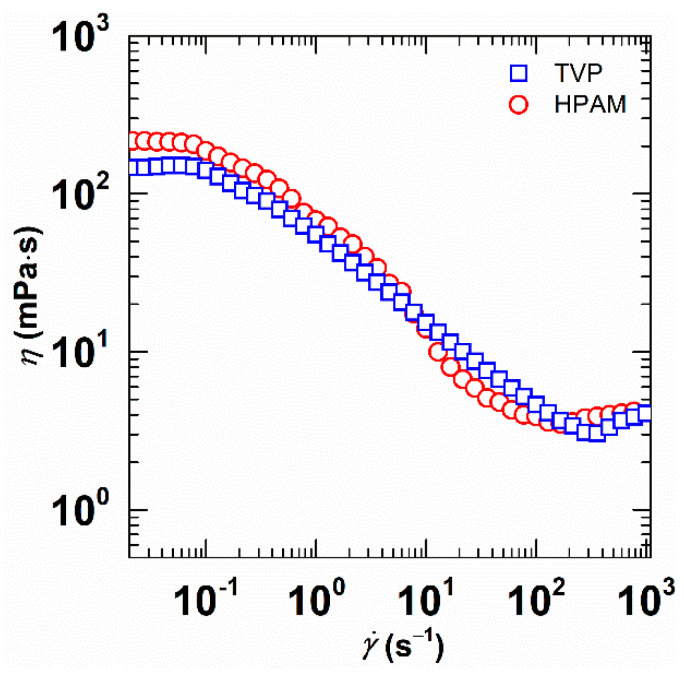
The apparent viscosity of 0.1% TVP and HPAM solutions with 950 mg/LTDS plotted against shear rate at 25 °C.

Compared in Figure 4 are the plots of the apparent viscosity of the 0.1% TVP and HPAM solutions prepared with 950 mg mg/L NaCl against shear rate from 0.02 to 1000 s^−^^1^. The first observation is that the two plots are quite similar: they can be divided into three parts—the first Newtonian plateau from 0.02 to around 0.1 s^−^^1^, the shear thinning region from 0.1 to 200 s^−^^1^, and the second Newtonian plateau from 200 to 1000 s^−^^1^. However, differences can also be noted, as the viscosity of the TVP solution in both the first and second plateau is slightly higher than that of the HPAM solution in the same region, but seems to decrease more rapidly than HPAM in the shear thinning region, i.e., the TVP solution is more shear-sensitive than the HPAM one, and the data points in the TVP plot are more scattered, in contrast to the smooth plot of the HPAM solution, which is indicative of associative aggregates existing in the polymer solution [27].

In general, the 0.1% TVP solution displays very similar shear flows to the HPAM solution; in particular, they have an almost superimposed shear thinning response, implying that TVP has comparable injection potential to HPAM.

### 2.4. Long-Term Thermal Stability

Normally, it takes more than half a year for the polymer solution slug to pass from the injection wells to the production ones, so it is vital to see if the apparent viscosity can be sustained after long-term aging during this transportation process, and to see if the remaining viscosity is sufficient to control the mobility of a polymer slug over a water one.

**Figure 5:**
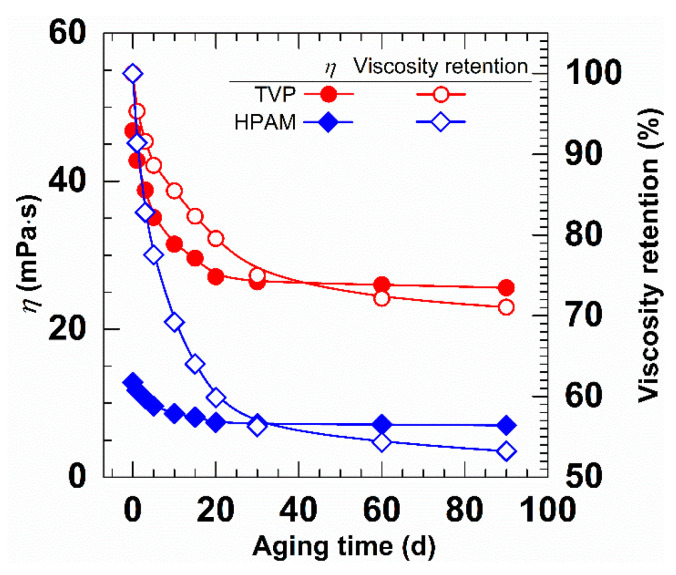
Viscosity loss and viscosity retention of 0.1% TVP or HPAM solution in the presence of 4500 mg/L TDS containing 65 mg/L Ca^2+^ and Mg^2+^ versus aging time. Shear rate, 7.34 s^−^^1^.

Compared in Figure 5 are the residual viscosity and viscosity retention for 0.1% TVP and HPAM solutions after different aging times under the simulated Daqing type III oil reservoir conditions. Both the TVP and HPAM solutions descend clearly over the aging time. After 20 days of aging at 45 °C, the viscosity of both polymer solutions stabilizes, and almost 37% of viscosity is lost for the HPAM solution, while only 20% of the viscosity is lost in the TVP solution. After three months of aging, the viscosity of the HPAM solution decreases to around 7 mPa⋅s, only 53% of its original viscosity; under the same condition, the viscosity of the TVP solution is 28 mPa⋅s, retaining 71% of its initial viscosity. Such comparison results clearly show that TVP has superior properties to HPAM for use in the displacement process under Daqing oil reservoir conditions.

### 2.5. Propagation of Polymer Solution in Porous Media

The success of polymer EOR projects depends on the ability of the injected polymer solutions to be transported deep into the reservoir, thus providing an enhanced mobility ratio for the displacement process [28]. A favorable mobility reduction could be achieved by a permeability reduction, a viscosity enhancement, or by a combination of the two. Thus, the injectivity of polymer solutions is a critical parameter and key risk factor for the implementation of polymer flood projects.

The propagation or transportation performance of polymer solutions in porous media is normally characterized by the resistance factor (*F*_r_) and residual resistance factor (*F*_rr_) [29]:

(1) $$F_{r}=\frac{{\left(\Delta P\right)}_{p}}{{\left(\Delta P\right)}_{\mathrm{wb}}}$$

(2) $$F_{\mathrm{rr}}=\frac{{\left(\Delta P\right)}_{\mathrm{wa}}}{{\left(\Delta P\right)}_{\mathrm{wb}}}$$

where (∆*P*)_p_, (∆*P*)_wb_, and (∆*P*)_wa_ refer to the pressure differentials during polymer flooding, water flooding before polymer injection, and water flooding after polymer injection, respectively. As can be seen from Equation (1), *F*_r_ represents the pressure increase of the polymer solution relative to brine, reflecting the capacity of mobility reduction by polymer flooding; from Equation (2), one can find that *F*_rr_ is defined as the ratio of pressure differentials before and after polymer injection, i.e., pressure caused by irreversible permeability reduction, indicating the permeability reduction caused by polymer flooding. Their values are always greater than 1.0, and larger values of *F*_r_ and *F*_rr_ indicate greater potential to improve the sweep efficiency and higher incremental oil recovery by polymer flooding.

**Figure 6:**
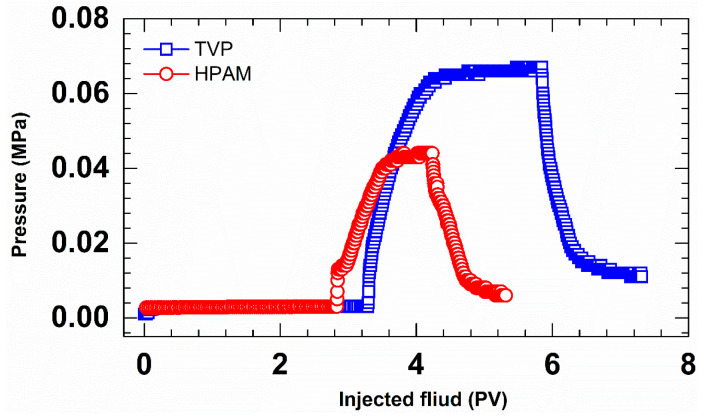
Variation in pressure versus pore volume (PV) during the propagation of 0.1% TVP or HPAM solution in natural cores at 45 °C with the injection rate of 2 mL/min. The 0.5% mother polymer solutions were prepared with 4500 mg/L synthetic produced fluid containing 65 mg/L Ca^2+^ and Mg^2+^, and then diluted to 0.1% polymer solution with the synthetic brine with TDS of 950 mg/L NaCl.

The pressure variations during the injection of TVP or HPAM solutions are depicted in Figure 6, and the results of *F*_r_ and *F*_rr_ calculated from Equations (1) and (2) are tabulated in Table 1. One can find that the *F*_r_ and *F*_rr_ values of 0.1% TVP are 50% and 85% higher than those of the HPAM solution with the same concentration and same salinity. The higher *F*_r_ value of TVP indicates that it has a stronger flow control ability over HPAM during the propagation process in porous media, and the elevated *F*_r_ arises mainly from thermoassocaition at high temperatures, thus resulting in higher viscosity (as can be seen in Figure 2); finally, its propagation through the porous media is slowed down and much higher flooding pressure is needed. Additionally, polymer retention may occur because of multi-layer adsorption from the associative polymer [30,31], building flow resistance in water-flooded zones and diverting the subsequent chasing fluid towards less swept, permeable zones.

The *F*_rr_ value of the 0.1% TVP solution is also higher than that of the HPAM solution, suggesting that TVP has a better ability to reduce the permeability due to polymer retention. Such a permeability reduction also partly originates from the retention of TVP, as aggregates with high hydrodynamic volumes (Figure 3) will be trapped or adsorbed by porous media more easily, shutting off the pores and reducing the permeability accordingly. The narrowed permeability difference between high- and low-permeability layers renders a more uniform frontier rather than “fingering”, thus promoting the sweep efficiency.

### 2.6. Enahnced Oil Recovery Efficiency

**Figure 7:**
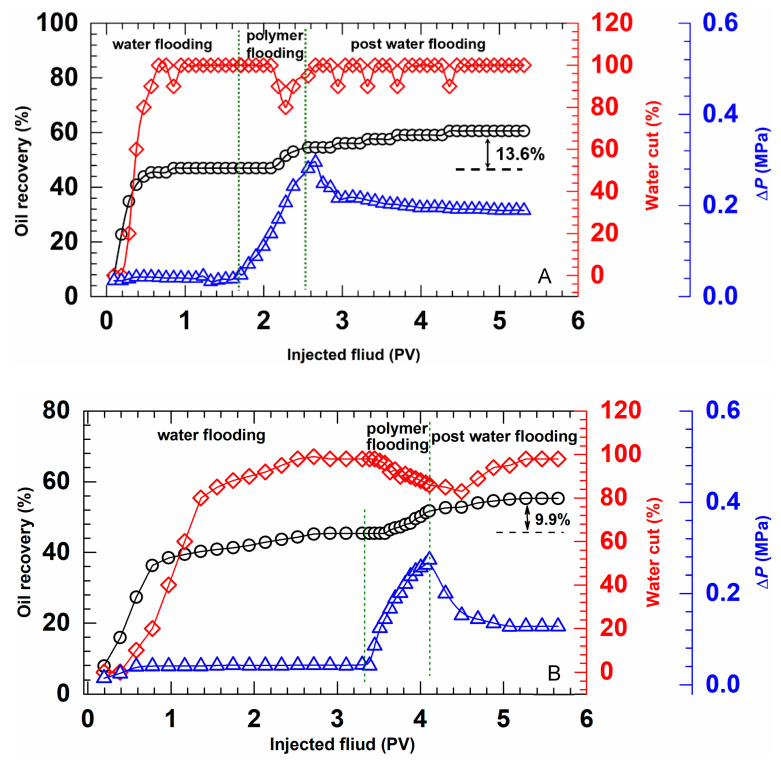
Variation in pressure and oil recovery factor for (**A**) TVP and (**B**) HPAM solutions during core flooding at 45 °C. The 0.5% mother polymer solutions were prepared with 4500 mg/L synthetic produced fluid containing 65 mg/L Ca^2+^ and Mg^2+^, and then diluted to 0.1% polymer solution with the synthetic brine with TDS of 950 mg/L NaCl.

While the basic properties of TVP are superior to those of HPAM, the key criterion for an EOR polymer is its enhanced oil recovery efficiency or oil recovery factor (E). Compared in Figure 7 are the variations in R_f_, water cut, and pressure differential (Δ*P*) versus the pore volume (PV) of the displacing fluids injected with the same polymer concentration of 0.1%, and the results are summarized in Table 2.

It can be seen in Figure 7 that the post-water-flooding pressure for both polymers is lower than that of the polymer flooding process, implying that both TVP and HPAM can be transported smoothly in the porous media with a water permeability of around 115 mD without plugging the cores. From the comparison in Table 2, one can find that after 1.7 PV and 3.4 PV water flooding for the cores No. 3 and No. 4, the recovery factor can reach 46.97% and 45.38%, respectively. Then, 0.7 PV of TVP and HPAM solutions were separately injected, and the water cut decreased from 98% to 80% for both cores. The total oil recovery factors (*E*_total_) of 60.61% and 55.28% (OOIP) are obtained, among which the contributions of TVP and HPAM (*E*_p_) are 13.64% and 9.90%, respectively. In other words, at an equivalent polymer concentration of 0.1%, TVP can recover 3.54% more oil than HPAM. The reason for the higher recovery factor of TVP lies in its abnormal thermoassociation behavior; thus, higher viscosity of TVP can be obtained in the reservoir conditions.

## 3. Materials and Methods

### 3.1. Materials

**Scheme 1:**
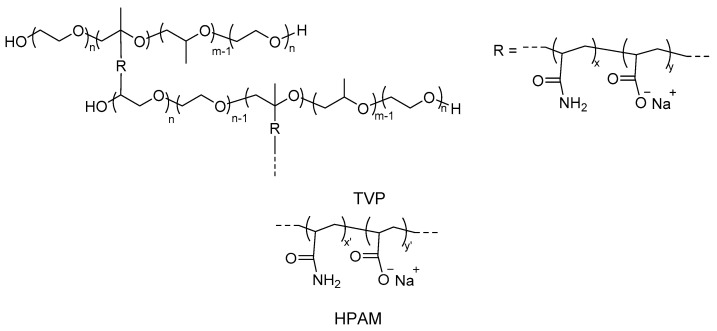
The molecular structures of TVP and HPAM used in this work.

The TVP was copolymerized with acrylamide, sodium acrylate, and Pluronic F127 with inverse emulsion polymerization, as reported previously [23]. The reference commercial polymer, HPAM, was provided by Daqing Refining & Chemical Company (Daqing, China). Their molecular structures are listed in Scheme 1, and their physical parameters are summarized in Table 3. The viscosity-averaged molecular weight (*M*_η_) of TVP and HPAM, estimated from their intrinsic viscosity [*η*] with the Mark–Houwink equation [*η*] = 9.33 × 10^−3^*M*_η_^0.75^ [32,33], was 7.6 × 10^6^ and 8.2 × 10^6^ g/mol, respectively. The degree of hydrolysis, i.e., the content of acrylate segment in the polymers, was 23.1% and 21.2% for TVP and HPAM, respectively.

The inorganic salts used, NaCl, CaCl_2_, and MgCl_2_, all of analytical grade, were provided by Chron Chemicals (Chengdu, China). Doubly distilled water with resistivity of 18.25 MΩ⋅cm was prepared using an ultrapure water purification system (Ultrapure Technology Co., Ltd., Chengdu, China).

The cores used were natural ones drilled from the type III oil layers in Daqing Oilfield. They were soaked and cleaned with solvent gasoline and then dried, and the gas permeability was determined under the condition of unchanged wettability.

### 3.2. Preparation of Polymer Solutions

As tabulated in Table 4, two types of saline water were used to prepare the polymer solutions, simulating the practical process where “mother solutions are prepared with fresh water, injected solutions are diluted with produced fluids”. The salinity (or total dissolved solids, TDS) of the first brine was 950 mg/L NaCl, which was used to simulate fresh surface river water, and the second brine was TDS of 4500 mg/L containing 4435 mg/L NaCl and Ca^2+^ and Mg^2+^ (65 mg/L), which was used to mimic the salinity of the produced fluids in Daqing Oilfield.

First, the synthetic brine was stirred with a propeller-type agitator at 500 rpm to generate a vortex of one-third of the total volume. Then, the designated amount of polymer powder or polymer emulsion was added slowly to the vortex shoulder. Next, the dispersions were stirred at 700 rpm for half an hour, followed by mild agitation at 200 rpm until complete dissolution. Then, the polymer solutions were aged for 1 day prior to tests. The polymer solutions used in propagation in porous media and in the enhanced oil recovery process were diluted with the synthetic produced fluid from 5000 mg/L of the mother solutions that were prepared with synthetic fresh brine. Other polymer solutions were directly prepared with the above brines to achieve target concentrations.

### 3.3. Viscosity Measurement

The apparent viscosity of all polymer solutions was measured with a Physica MCR 302 (Anton Paar, Graz, Austria) rotational rheometer equipped with a Searle-type concentric cylinder geometry CC27. The radii of the measuring bob and the measuring cup were 13.33 and 14.46 mm, respectively. The polymer solutions were equilibrated at designated temperatures for around half an hour before measurement. Then, the steady shear apparent viscosity (*η*) was recorded at a fixed shear rate ($\dot{\gamma }$) of 7.34 s^−1^. For the temperature scan, the heating rate was fixed at 2 °C/min. The temperature was controlled by a Peltier system, and a solvent trap was used to prevent the evaporation of the solvents during measurement.

### 3.4. Determination of Hydrodynamic Diameter of Polymers

The hydrodynamic diameter (*d*_H_) of both TVP and HPAM was determined using a Zetasizer Nano ZS (Malvern, UK). The 0.1% polymer solutions were prepared with the synthetic produced fluids as the solvent, and the prepared solutions were filtrated through a syringe filter with a pore size of 0.22 µm to remove impurities. Then, the polymer solution was placed in a disposable capillary cuvette and was measured three times to obtain an average value. The temperature was controlled by an integral Peltier device over the range of 20 to 50 °C with the precision of ±0.1 °C.

### 3.5. Long-Term Thermal Stability

Following the enterprise standard (Q/SY 1192007) [34], both TVP and HPAM were examined comparatively concerning their application properties, targeting polymer EOR. The polymer solutions used for long-term thermal stability monitoring were prepared in a Model-2000 vinyl anaerobic glove box (Coy Lab Instrument Co., Grass Lake, MI, USA). The box was evacuated and then re-streamed with high-purity nitrogen several times to ensure that the final oxygen content in the glove box was kept below 10 ppm. Two-hundred-milliliter polymer solutions were transferred into different steel cans, which were sealed and aged at 45 °C in a thermostatic oven. At a consecutive time interval, the polymer solution was taken from one can, and its viscosity was measured with a Brookfield DV-II viscometer.

### 3.6. Injectivity

The length and diameter of the reservoir core were measured before placing them into the core holder. The core was vacuumed for 2 h at the confining pressure of 3 MPa, followed by saturation of the synthetic produced fluid until the reading in the pressure gauge returned to zero. The porous volume (PV) and porosity (φ) of the core were calculated according to the volume of synthetic produced fluid injected. Then, the core was flooded by the synthetic produced fluid at different injection rates, and the pressure differential was recorded to calculate the permeability (K) of the core according to Darcy’s law. Next, the core was flooded by synthetic produced fluid and polymer solutions to carry out water flooding, polymer flooding, and post-water flooding.

### 3.7. Core Flooding Test

Following our previously reported procedure [35], here, we simply describe a typical process to calculate the recovery factor (R_f_). The parameters K, φ, and PV were determined with the same procedure as described in Section 3.6. Then, the cores were initially saturated with synthetic produced brine under vacuum, followed by injecting the mixed crude oil with viscosity of around 10 mPa·s to set the initial oil saturation until the water cut was less than 2.0%. Water flooding was first carried out until the water cut of the produced fluid reached 98%; then, polymer flooding was conducted with the injection of a 0.7 PV displacement plug, and post-water flooding was carried out until the oil production became negligible. During the flooding process, the injection rate of the displacing fluids was controlled at 2 mL/min and the test was run at 45 °C for 0.1% TVP or HPAM solution, respectively. The incremental oil recovery by polymer slug injection and the post-water flooding was used to evaluate the efficiency of these systems. The pressure drop across the core during the flooding was also monitored.

## 4. Conclusions

Although HPAM has been widely used in the chemical EOR process, the drawbacks of thermothinning and poor salt tolerance have impeded its use in low-permeability oil reservoirs. In this work, we compared the basic properties and oil recovery efficiency of a thermoviscosifying polymer versus HPAM with similar molecular weight and hydrolysis degree under the simulated conditions of the type III reserve in Daqing Oilfield.

At 25 °C, TVP and HPAM have comparable viscosity in 950 mg/L NaCl, but the former shows much stronger thickening power at 45 °C, even though the brine is more saline. At 0.1% polymer concentration, HPAM shows typical continuous thermothinning behavior in both synthetic surface brine and synthetic produced fluid, but TVP shows abnormal thermothickening from 40 to around 55 °C after a slight decrease from room temperature to 40 °C in both brines. The temperature of the type III oil reserve of Daqing Oilfield falls within such a viscosity enhancement range. After 90 days of thermal aging at 45 °C, TVP can maintain 71% of its original viscosity, 18% higher than that of HPAM. Although both polymer solutions can smoothly pass through the porous media, TVP possesses a stronger mobility reduction and permeability reduction capacity. In addition, 0.1% TVP can obtain an oil recovery factor (OOIP) of 13.64%, 3.74% higher than HPAM under identical conditions.

In short, TVP exhibits superiority over HPAM because of the unique thermothickening behavior, and the above basic and application properties indicate its great potential for use in low-permeability oil reservoirs and field trials where polymer mother solutions are prepared with fresh water but diluted with more saline produced fluids.

## Figures and Tables

**Table 1:** The resistance factor (*F*_r_) and residual resistance factor (*F*_rr_) of 0.1% TVP and HPAM solutions flowing in natural Daqing cores at 45 °C.

Core No.	Polymer	Length (cm)	Diameter (cm)	PV (cm^3^)	*φ* (%)	*k*_w_ (mD)	*F*_r_	*F*_rr_
1	TVP	7.24	2.50	8.52	23.97	114.40	21.61	3.87
2	HPAM	7.55	2.49	8.85	24.07	104.90	14.67	2.00

**Table 2:** The core flooding results of 0.1% TVP and HPAM solutions at 45 °C.

Core No.	Polymer	Length (cm)	Diameter (cm)	PV (cm^3^)	*φ* (%)	*K*_w_ (mD)	*E*_w_ (%)	*E*_total_ (%)	*E*_p_ (%)
3	TVP	8.76	4.84	11.11	26.22	141.7	46.97	60.61	13.64
4	HPAM	8.03	4.96	10.56	26.53	115.2	45.38	55.28	9.90

**Table 3:** Physical parameters of TVP and HPAM used in this work.

Polymer	Form	*M*_η_ (×10^6^ g/mol)	Degree of Hydrolysis (mol %)
TVP	Emulsion	7.6	23.1
HPAM	Powder	8.2	21.2

**Table 4:** The composition and salinity of the synthetic fresh brine and produced fluid.

Component	Synthetic Fresh Brine (mg/L)	Synthetic Produced Fluid (mg/L)
Na^+^	374	1692
Ca^2+^	None	50
Mg^2+^	None	15
Cl^−^	576	2743
Total dissolved solids (TDS)	950	4500

## Data Availability

The data presented in this study are available on request from the corresponding author.

## Sample Availability

Samples of the compounds, including TVP and HPAM, are available from the authors.
